# A Letter to Sir Henry Halford, Bart., in Which an Account Is Given of a Severe Epidemic Disease of the Stomach and Bowels, That Occurred at Douglas, Isle of Mann, in August and September Last

**Published:** 1832-01

**Authors:** H. R. Oswald

**Affiliations:** Surgeon to the Household, Isle of Mann.


### EPIDEMIC DISEASE OF THE STOMACH AND BOWELS.

*A Letter to* Sir Henry Halford, Bart., *in which an Account is given of a severe Epidemic Disease of the Stomach and Bowels, that occurred at Douglas, Isle of Mann, in August and September last.* By H. R. Oswald, Surgeon to the Household, Isle of Mann.

Sir: Having perused the papers relative to the disease called Cholera Spasmodica, published by the Board of Health in London, and signed by you, Sir, (with which I was obligingly favored by Robert Stewart, Esq., collector of this port,) and in which the Board invites correspondence on the subject, I consider it my duty, during the non-existence of a local Board in this island, to address the following notices to the Board of Health relative to a severe disease of the stomach and bowels, which has been epidemic in this town and neighbourhood for five weeks past. The epidemic has always been very general in this town, which contains about seven thousand inhabitants.

Few families have escaped, having one, two, or more of their members subjected to it. The cases have assumed a great variety of aspects and modifications of symptoms, from very mild to very severe, so much so that we might question the identity of the disease in all cases being the same. No arrangement has been adopted to stop its progress: indeed, this step does not now seem to be so necessary as it was, not only because the course of the disease appears to be nearly at an end, but because, though very general, and in some cases very dangerous, it has not excited any very striking alarm among the inhabitants, perhaps owing to its proving so little fatal. In this notice I simply call it a disease of the stomach and bowels, and do not attempt to arrange it under any nosological name; only give a history of several well-marked cases, severe as well as mild ones, and in some in the patient's own terms, in order that the Board may themselves determine what name to call it by; for it would be worse than erroneous in me to call it by a name which might excite alarm, especially if that alarm turned out to be unnecessary.

During the months of June and July, and previous to the appearance of this disease, or at least previous to its becoming general, we had been visited by what was called "the influenza," a catarrhal disease accompanied by symptoms of the Synochus of Dr. Cullen, which was ephemeral in its duration, or seldom lasted beyond two or three days, and which proved fatal in a few cases that were protracted into continued fever. Besides the catarrhal symptoms, which often attracted little notice, the leading symptoms of this fever were high vascular excitement, ardent heat of surface, palpitation at the heart, headach more or less intense in some, or vertigo in others; with great prostration of strength, lassitude, tongue white and loaded, and brindled as it were with white scurf upon red; severe local pains in the trunk of the body at various points, and generally with hurried respiration.

1. In one case, which proved fatal in a very healthy middle-aged woman, after running a course of four weeks, the pain was in the right hypochondriac region, accompanied by cough. In this case the patient was recovering from the eighth to the fourteenth day, but she then suffered a relapse of her illness, accompanied with frequent spasms in the stomach, and bowel complaint. She said there was something in her stomach she could not get rid of; she had constant tendency to sleep during the last week of her illness, but slept badly, and at length died comatose on the 5th of July, twenty-eight days after the first attack.

2. On the 18th of July, Mrs. H. was seized with symptoms of the influenza fever, which not subsiding after the first few days, were cut short by the usual means: "from which," (I quote the lady's own account,) "I had recovered so as to be out, but, on the 4th of August, about two o'clock in the afternoon, I was seized with giddiness in my head and dimness of sight, alternately burning with heat to a sense of suffocation, and then shivering from head to foot, with a spasmodic pain in my bowels, with nausea, but no vomiting or purging. I became excessively thirsty, with a burning heat at my stomach and violent throbbing at the heart; my tongue was foul, and I had the taste of copper in my mouth; I felt a sensation of illness I can give no adequate idea of. Towards evening my body became enlarged, with a settled pain in the lower part. I went to bed at nine," (having tried to eat some cold salmon and vinegar for supper,) "but my head ached so violently, my feet were so cold, and my eyes so painful, I could not sleep. About two o'clock I was roused from my bed by what I supposed a violent bowel complaint; the evacuations continuing so rapid, violent, and painful, I could not rest a moment in bed. About four I became worse, was parched with thirst, vomiting, powerless, had a dreadful sensation at the back of my head, like the sound of a water-wheel; my own voice startled me; I felt horror-struck; my eyes seemed drawn to the back of my head, and I was instantly seized with violent cramps in my hands, legs, thighs, and feet, when I attempted to stand and press with my feet, in order to counteract the cramp. I fell from one fainting fit into another; (I am told my countenance now had the hue of death;) still suffering pain all over me; the evacuations continued at this period almost unconsciously; I was exhausted; I could not raise my head, and believed myself dying. At about half-past five Mr. Oswald was sent for; he came immediately: I could then scarcely raise my eyelids, and shall never forget my sensation of horror when, pressing my fingers upon them, I could not feel my eyes; they were burning, and apparently fixed, for I could not move them, nor could I bear the light during that day. I felt almost immediately relieved by the medicines administered by Mr. Oswald. The sensation in my eyes the evening previous to the attack, was of pins or needles running into them."

The foregoing, though a lively, is a true state of the case of this lady. When called, I found her surface cold to the sensation; expression of countenance pallid and shrunk; eyelids half closed; complaining with a hurried tremulous voice; and unable to rise from bed, anxiously pressing her feet against the cold wall of the room, for relief from the spasms in the legs; pulse a thread at the wrist; rejecting every thing from the stomach immediately; with incessant alvine evacuations of a greyish serum; urine scanty, and passed with pain. After recovering, she experienced no relapse, and is again quite well.

3d. On the night of Monday, August 1st, a case precisely similar, but less lively in description, occurred in a stranger at one of the inns, except that he complained of no headach; who insisted next morning on being able (but with much lassitude and languor in the eyes,) to proceed homewards in a voyage to Liverpool.

4th. On the night of the 5th of August, Mrs. P. was seized: she had no cramps in the legs or hands, but very severe spasms in the abdomen, with incessant vomiting and purging for five hours.

5th. On the night of the 6th, Mrs. B. had no pain in the stomach or bowels, but incessant purging and vomiting of a brownish grey serum, and severe cramps and muscular contractions in the calves of the legs. This case lasted nearly twelve hours, and had less tendency to collapse than might have been expected from the long duration of the evacuations and pain. She recovered slowly, owing to a tendency to bowel complaint, but had no secondary fever.

6th. About seven o'clock in the morning of the 8th of August, Mr. Charles Y., a young man, was taken with symptoms "which" (I quote his own words,) "manifested themselves in excessive thirst, nausea, vomiting, and purging, but unaccompanied with any griping pain in the bowels or pain in the head or eyes; yet felt a good deal of faintness and giddiness. The complaint gained ground, and was attended with the most excruciating cramp and convulsions in the feet, legs, and thighs, with sensation of extreme coldness in them. The cramp continued for the space of four hours at intervals of ten and fifteen minutes, but was at length subdued by the warm bath and other remedies. The purging ceased from the time I obtained relief from the cramps, but the nausea and vomiting continued at intervals throughout the day. The next day I was a great deal relieved, but left in a very weak and wearied state of body," with soreness and tenderness where the cramps had been.

7th. On Tuesday the 9th, Mrs. H., who had suffered from bowel complaint since Saturday the 6th, which she ascribed to fatigue during that hot day, was seized with incessant vomiting and frequent and exhausting alvine dejections of brownish grey-coloured serum; urgent thirst; wiry and hard pulse, but regular and beating about one hundred; no pain or spasms in the bowels or in the extremities, which are also warm, but has frequent attacks of an intolerable spasm in the back, just inferior to the right scapula. This case was not relieved for nearly twelve hours; every thing was rejected from the stomach; and it was the only case in which I considered it necessary to employ venesection.

8th. During the night of the 9th, Mr. H., an elderly man, had a very severe attack: when I saw him the following day, his pulse could just be distinguished, beating like a thread irregularly at the wrist; extremities cold, and could not be kept warm by the application of heat; and he was incessantly calling out, in a hoarse, whispering, querulous tone, that the cramp was now in his hands and fingers, and now in his feet and toes, in order that he might get relief by rubbing with the warm hand of the attendant; a considerable degree of collapse was expressed in his countenance, and his eyes were half shut. Reaction took place in the afternoon, and he recovered without any secondary symptoms.

9. On the 10th, Mrs. N., with whom Mrs. H., No. 5, lodged, began to complain of pain in the bowels, with exhausting diarrhœa, which symptoms continued to reduce her, sometimes better, sometimes worse, and sometimes with tenesmus and bloody and mucous evacuations, till the beginning of September.

10. About the middle of August, the complaint, in the form of a griping diarrhœa, or short attack of vomiting, with frequent liquid motions, without any other bad symptoms, became very frequent. In my own family, a little boy, twenty months old, suffered from the diarrhœa for three weeks; his stools often loaded with mucus, and sometimes of an orange colour, and a green mixed with blood. I remarked the same appearances in the stools of many other children, who had been ill for a week or two.

11. On the night of the 12th I experienced an attack myself: I retired to rest, feeling even better than usual, and was awakened suddenly, about three o'clock in the morning, by pain in the abdomen and bowel complaint, of uncommonly copious and frequent evacuations, without nausea or vomiting; after about an hour, a severe cramp of the muscles of the calf of the left leg only seized me, which induced me to call for assistance: it continued to recur at intervals for some hours.

Three hours after the attack, I felt slight nausea, and threw up a mouthful or two of dark-coloured mucus; and the evacuations continued, but were simply a watery serum, passed copiously and forcibly without much pain, but producing rapid prostration of strength and sinking in the tone of voice. My extremities were not cold; I had little or no thirst; pulse hard, but little accelerated; and there was a sensation of pricking stiffness or rigidity in the eyelids. Five hours after the attack, the pain and other symptoms had ceased, and I only experienced considerable prostration of energy and a disposition to sleep. The cramp in the leg seemed to alternate with pain in the abdomen; that is, when pain was felt in the abdomen, none was felt in the leg, and *vice versâ.* Next day the muscles of the leg, where cramp had been, felt very tender.

12th. About a week after this, another of my family experienced an attack of the intolerable nausea and vomiting, which she described as exactly resembling severe sea sickness, with acute pain at the scrobiculus cordis, coming on in spasms, at intervals of about ten minutes, but unaccompanied with purging or with cramp in the legs; and which lasted for six or seven hours.

The nurse had symptoms of its approach the following day, but was immediately relieved by an emetic.

About the middle of August it was common to hear some of the inhabitants say that such a person had an attack of the native or Manx cholera; on what authority I could not learn.

13th. Mrs. G., aged seventy-four years, applied to me on August 24th, on account of symptoms of continued fever and prostration of strength, with pain, feeling of distention, and uneasiness of the bowels, which had confined her to bed for a week. Pulse full and 100; tongue rather furred, and loaded with mucus; no tension in the abdomen or pain on pressure there; moans and complains querulously of a general sensation of distress; bowels not too open for the first few days. During the last three days of her illness, she had frequent watery motions, which she passed often unconsciously; said there was a feeling of some oppression about her stomach she could not get rid of; mind collected when roused, but there was a tendency to coma, and sometimes muttering incoherence of speech, with an anxious expression of countenance, and a dark circle about the eyes; which, however, was her natural expression. She died on the tenth day of the disease.

14th. On the 7th of September, Thomas S., aged fifty, labourer, was attacked with pain in the abdomen, a burning sensation at the pit of the stomach, with urgent thirst, about three P.M. Soon after, great failing of the strength, incessant vomiting and purging, and severe cramps both of the upper and lower extremities, came on, without any relief to the pain at the scrobiculis cordis. I saw him at six o'clock P.M.: all these symptoms were in full force; surface felt cold, and pulse nearly gone at the wrist; his respiration quick and anxious; collapse seemed to be advancing, and he anxiously rolled his head on the pillow from side to side, complaining that, besides the cramps, he felt uneasy and very ill all over. His alvine dejections were entirely serous, but, after reaction took place, they assumed a yellow colour. He obtained relief between seven and eight o'clock. During the night he had some refreshing sleep, and next day was so much better that he talked of going out.

Such is a sketch of the leading features of the disease that has prevailed here for the last five weeks or more. Much of the variety that occurred in the cases will be manifest from it. The great majority of the cases were so mild that the patients did not require more medicine than a dose or two of castor oil or other aperient. In the mild cases there was little or no pain. In severe cases there were spasmodic pains, not only in the abdomen and extremities, but all over the body, drawing the muscles into knots and contractions. Sometimes there was pain in the limbs, and none in the abdomen, or in the muscles of the trunk of the body, and *vice versâ*. Sometimes there was no vomiting; sometimes no purging; sometimes the patient was laid entirely prostrate in half an hour; whilst in others he continued to go about. In some cases, the giddiness was severe, and caused instant nausea on attempting to move; in others, it was trifling, and only felt at intervals. In some cases there was a tendency to sudden collapse and coldness of the extremities; whilst in others the fever ran high, and the heat of the whole body was oppressive. Sometimes there was no fever, excepting a quickness of pulse and an excitement arising from suffering, pain, and nervous irritability. The tongue, in general, presented nothing very peculiar; it was often white in the middle with tendency to dryness, sometimes covered with white scurf brindled on red, according to the state of the habit and the stage of the disease; the stools were feculent, serous, bilious, mucous, or bloody. In other cases there was none of all these; nothing but an inactive and painful state of the bowels and stomach; a sensation as if the abdomen was distended and the body enlarged; in some cases contractions of abdominal muscles; palpitation at the heart, and vertigo; with loss of appetite, much flatulence, and uneasy sensations, as if in want of medicine. Urine was generally scanty, but not always; pulse often hard, sometimes small and irregular.

In some the duration of the disease, though very severe, was short, not exceeding six or eight hours; in others it continued as in Mrs. N., No. 7, hanging about the patient for weeks, undergoing frequent and severe exacerbations. In the short attacks it was always more severe for the time; and in many cases, though not in all, left a difficulty in regulating the bowels, and a necessity for caution in regimen, to guard against a relapse; in others the recovery was complete, and the health was better than previous to the attack.

According to my observation, the symptoms most constantly present in every case were more or less giddiness of the head, and a failing of the strength, or debility almost to faintness; also often a rigidity of the muscles of the eyelids, or pain and uneasiness in the eyeballs: nothing relieved these symptoms more than sleep. There can be no doubt that the brain and nervous system were more or less affected in every case; although, in the very worst stages of the worst cases, the mental functions remained collected and available. Even Mrs. G., No. 11, although labouring under severe secondary fever, with a tendency to coma, could be roused to consciousness. It may be questioned whether this case, as well as No. 1, were identical with the disease which I intend at present to describe, the most marked cases of which they were so very unlike. At all events, they were cases of the influenza fever, and they appeared to me mixed cases of the influenza and the spasmodic disease: this combination seemed the cause of the difficulty and danger experienced in these cases. The influenza fever was certainly the most dangerous of the two diseases. Where there was no fever of a dubious bad type, and we had only to contend with the spasmodic disease, the case was much more tractable.

It was only such compound cases that proved fatal, and, as in these cases death was the consequence more of the influenza than of the spasmodic disease, it becomes a question whether even one case of the latter proved fatal. Upon referring to the registry of burials for the last six weeks, I find this remark to be correct so far as relates to grown-up persons. As the disease, if it affected children at all, was very different in them, it remains doubtful whether we ought to refer any cases of bowel complaint that proved fatal amongst them to the spasmodic disease.

During a residence and a practice here of nineteen years,

I have not observed any disease similar to that just described. Affections of the bowels are frequently prevalent: in autumn 1826, a disease of this kind, of a dysenteric tendency, prevailed, and proved fatal to several aged persons; but throughout its course no cases occurred with spasm or cramp in the external muscles. There are no data to ascertain the number attacked; no municipal arrangement of that kind exists in the Isle of Mann.

As I have already remarked, no simple attack of the spasmodic disease proved fatal: I am unable to ascertain how many died of the fever. The disease is now much diminished in frequency. Appearances of blood in the stool have become more common. The last severe case was that of Thomas S., No. 12, and a few milder cases are still occurring.

I shall conclude this report with these remarks, and, with reference to the plan of cure, shall wait another opportunity, when, if it is the pleasure of the Honourable Board, I shall intrude further on their patience.

I have the honour to be, Sir, your obedient humble servant,

(Signed) H. R. OSWALD,
Surgeon to the Household, Isle of Mann.

*September* 10, 1831.

To Sir Henry Halford, Bart.
Chairman to the Board of Health, College of Physicians, London.

